# Evaluation of knowledge of risk factors and warning signs of stroke – An observational study among future health care professionals

**DOI:** 10.3389/fpubh.2023.1131110

**Published:** 2023-03-08

**Authors:** Wajid Syed, Omaimah A. Qadhi, Amal Barasheed, Ebtesam AlZahrani, Mahmood Basil A. Al-Rawi

**Affiliations:** ^1^Department of Clinical Pharmacy, College of Pharmacy, King Saud University, Riyadh, Saudi Arabia; ^2^Medical-Surgical Nursing Department, College of Nursing, King Saud University, Riyadh, Saudi Arabia; ^3^Department of Optometry, College of Applied Medical Sciences, King Saud University, Riyadh, Saudi Arabia

**Keywords:** risk factor, pharmacy students, warning signs, stroke, health care professionals, nurses

## Abstract

**Background and objective:**

The role of healthcare professionals in society is unique since they are providers of health information and medication counseling to patients. Hence, this study aimed to evaluate Knowledge of Risk Factors and Warning Signs of Stroke among undergraduate health care Students (UHCS) at King Saud University (KSU), Riyadh, Saudi Arabia.

**Methodology:**

An online cross-sectional study was conducted among UHCS at KSU, Riyadh, Saudi Arabia from September to November 2022, using self-administered 34-item questionnaires divided into five sections to assess participants' knowledge of stroke risk factors, warning signs, and management and source of information about the stroke. The Statistical Package for the Social Sciences version 26 was used to analyze the data (SPSS).

**Results:**

Of the 300 questionnaires distributed, 205 students completed the questionnaires, giving a response rate of 68.3%. Of whom 63 (30.7%) were pharmacy, 81 (39.5%) were nursing and 61 (29.8%) were emergency medical services (EMS) Students. One hundred and eighty-two (88.8%) of the students agreed that stroke affects bodily movement. With regards to risk factors, students identified high blood pressure 182 (88.8%), followed by heart disease 175 (85.4%), advanced age 164 (80%), previous Stroke history 158(77.1%), and lack of physical activity 156 (76.1%). Difficulty in speaking or slurred speech 164 (80%), dizziness, and loss of balance 163 (79.5%) were identified as the warning signs of stroke. In this study, 41.3 % of the pharmacy students reported a good level of knowledge than nursing and EMS students. However, 32.2% (*N* = 66) of the healthcare undergraduates reported good knowledge. The knowledge score was significantly associated with the year of study, and educational degree (*p* = 0.0001). Furthermore, there were no differences between parents working in healthcare settings (*p* = 0.99).

**Conclusion:**

In conclusion, the knowledge of stroke among healthcare students at King Saud University varied. The reported knowledge gap mostly relates to stroke risk factors and warning signs. Therefore, increasing public awareness of potential risk factors and stroke warning signs needs to receive more attention.

## Introduction

A stroke is often described as a brain attack and a cerebral accident (1). It's a medical emergency that occurs when the blood supply to the brain is interrupted. Brain cells start to degenerate in minutes (1). Stroke is a chronic disease that affects people of all races and all generations (1, 2). It is currently regarded as a worldwide health problem that causes functional impairment and mortality. Stroke prevalence has risen recently on a regional and international level, becoming a major public health concern that is anticipated to keep getting worse (1, 2). The WHO estimates that 70% of strokes, and 87% of stroke-related deaths and disability-adjusted life years, occur in low- and middle-income countries (2). Strokes were challenging to recover from strokes can be severely disabling. It is evidenced that the incidence of stroke-related complications increases treatment costs, repeated visits to clinics, disability, and early mortality (3, 4). Stroke has become a significant and growing problem mostly due to unhealthy food habits, lack of physical activity, uncontrolled urbanization, and sedentary western lifestyles all of which contribute to multiple comorbidities (2, 3).

In Saudi Arabia, studies indicated that the number of stroke-related fatalities is on the rise, with an estimated number of Saudis dying each year from stroke (3, 4). According to the WHO, stroke is the second leading cause of stroke-related impairments globally (3, 4). In recent years the prevalence of stroke has been increasing and emerging as a major health problem, and it is estimated that the mortality rate resulting from stroke would be doubled by 2030, in Saudi Arabia (5, 6). These numbers indicate that stroke will have a great economic burden in Saudi Arabia in the future. Earlier literature in Saudi Arabia revealed that hypertension and smoking age were the most common risk factors for stroke (5, 6).

Besides its complications, the prevalence of stroke is proliferating in both developed and developing countries worldwide (7). It is evidenced that the incidence of stroke can occur in people over the age of 65 and they can occur in much younger ages (7–10). Healthcare students must be aware of the clinical presentation of various diseases since this information may be useful to them when they begin practicing after graduation. It was evidenced that healthcare undergraduates reported variation in their knowledge about stroke (11–13). For example, earlier findings revealed that nursing students have good knowledge of some aspects of warning signs and risk factors for stroke (11). Similarly, another recent study among university students reported an incomplete understanding of the risk factors of stroke (12). On the other hand, a previous study among Saudi medical students in Saudi Arabia revealed sub-optimal knowledge of ischemic stroke (13).

Although it is generally known that today's undergraduates would become tomorrow's professionals, a thorough awareness of the clinical facts related to diseases will help them in their work and help them to give their patients the best care possible. (14–19). Additionally, awareness of stroke would have a significant impact on morbidity and mortality rates, as well as contribute to the promotion of healthy habits. To increase students' knowledge, attitude, and practice (KAP) regarding stroke, a more extensive education program is required. Earlier studies have examined students' understanding of and awareness of stroke up to this point (11–13). To the best of our knowledge, there is a dearth of literature about clinical presentations and awareness of stroke among the UHCS in Riyadh Saudi Arabia. Hence, such a study was required and would help in future research. This study aimed to evaluate Knowledge of Risk Factors and Warning Signs of Stroke among UHCS in KSU, Riyadh, Saudi Arabia.

## Methods

### Study design and settings

We conducted a cross-sectional paper-based survey study among male students in healthcare colleges at KSU, including the College of Pharmacy, Nursing, and Emergency medical services (EMS) between July 2022 and October 2022, over four months. The male undergraduates aged >18 years and older, who were willing to complete the questionnaires and currently enrolled in the courses, and undergraduates with regular visits to college were included. Before accessing the survey, a disclosure statement followed by consent and agreement to use filled-out information for publication purposes was highlighted. We excluded students from other disciplines. Furthermore, the study was approved by the ethics committee of the College of Medicine at King Saud University. Before data collection, informed consent was obtained from the participants. Respondent's anonymity and confidentiality were ensured throughout the study.

#### Sample size estimation

There were ~350 residential students currently enrolled in Pharmacy, Nursing, and emergency medical services in the third and fourth years of courses at the KSU campus. Similar to the previous studies we calculated the required sample size using an online calculator (20–23) (http://www.raosoft.com/samplesize.html) with a 95% CI and a pre-determined margin of error of 5%. Because we were unaware of the potential results for each question, we assumed that the response distribution for each question would equal 50% (22). Although the sample size was projected to be 184, we opted to poll at least 300 students to assure greater reliability.

### Questionnaire design

In this study, we developed a questionnaire based on previous research about the knowledge of stroke risk factors and warning signs among undergrads at health colleges (11–13). The questionnaire consisted of 34 questions divided into five categories. In the first section, there were a total of six questions about the student's background, including the type of health, college, year of study, and knowledge of stroke (3 items). The second and third sections include knowledge of risk factors and warning signs of strokes. All these questionnaires were graded on a three-point scale (Yes/No/I don't know), and the fourth section discusses the management of stroke with a total of 5-items, assessed on a binary scale (Yes/No), the last section of the study questionnaires ask participants about the sources of information for stroke on a multiple choice. With the assistance of two prominent professors, the questionnaires underwent accuracy and content checks after initial compilation (one from the college of pharmacy and one professor from the college of nursing). An anonymous sample of students (*n* = 30) was surveyed for a pilot study. Pilot study results were not included in the final analysis. The reliability of the questionnaires was calculated by assessing the Cronbach's Alpha value (0.75) of the questionnaires, indicating it was reliable to carry out the study.

In the survey, nursing, EMS, and pharmacy students who were regular students at the colleges were included. During lecture periods, a researcher who was designated to collect data visited the students in their classrooms. A brief presentation was given to explain the purpose of the study and to assure students that their responses would be kept confidential. The students provided written informed consent. The questionnaire was given to participants with sufficient time to complete it. Data was collected using convenience sampling. Students who did not complete more than half of the study questionnaires were considered to have incomplete responses and were therefore excluded from the study, whereas students who did not complete 2 or 3 items in the survey were considered to have a treatable response and were thus included in the study. Non-respondents were students who did not return their questionnaires. The stroke knowledge score was computed by assigning a score of ‘1' for the correct answer, and a score of ‘0' for the wrong answer, likewise the total knowledge score was designed by computing the total knowledge items, which was further divided into good knowledge scores (who score of >50%) while poor knowledge score (a score < 50%) of the total score.

### Statistical analysis

An evaluation of the data was conducted using the Statistical Package for Social Sciences (SPSS) version 26.0 software. Descriptive analysis such as frequencies (n) and percentages (%) were assessed. The knowledge score and standard deviations (SD) were calculated and presented in the form of tables and graphs. In addition, the associations between categorical variables were determined by performing chi-square and Fisher exact test. A *p*-value < 0.05 was considered statistically significant.

## Results

### Demographic information

Of all participating subjects (*n* = 205), 63 (45.3%) were pharmacy students, 81 (54.6%) were nursing students and 61 (29.8%) were EMS Students. Most of the respondents were between 18 and 22 years of age. Only 27.3 % of students' parents work in a healthcare setting. Hundred and eighty-two (88.8%) of the students agreed that stroke affects bodily movement, and the majority 93.7% of pharmacy 88.9% of nursing, and 91.7 % of EMS students, reported that stroke happens when blood flow to the brain stops. While One-third (38.5%) of the students reported, the window period of thrombolysis was between 0 and 4.5 h. The detailed responses were presented in Table 1.

**Table 1:** Demographic characteristics of the participant.

**Variables**	**Frequency *n* (%)**
**Educational degree**	
Pharmacy	63 (30.7)
Nursing	81 (39.5)
EMS	61 (29.8)
**Do any of your parents work in a healthcare setting**	
Yes	56 (27.3)
No	149 (72.7)
**Level of education**	
Third year	108 (52.7)
Fourth-year	97 (47.3)
**A stroke or brain attack happens when blood flow to your brain**	
**is stopped**	
Yes	188 (91.7)
No	05 (2.4)
I don't know	12 (5.9)
**A stroke affect ability to move eat and other body function**	
Yes	182 (88.8)
No	08 (3.9)
I don't know	15 (7.3)
**What is the window period of thrombolysis in hours?**	
0–4.5 h	79 (38.5)
4.5–6 h	78 (38.0)
12–24 h	33 (16.1)
>24 h	15 (7.3)

### Knowledge of risk factors and warning signs among participants (*n* = 205)

**Figure 1:**
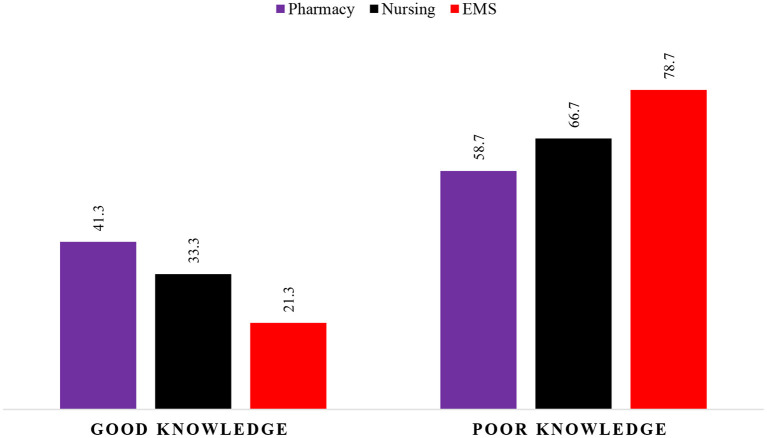
Level of knowledge according to educational degree.

Of the participants, most of them (88.8%) identified high blood pressure as one of the most common risk factors for stroke, followed by heart disease (85.4%), advanced age (80%), previous Stroke history (77.1%) and lack of physical activity (76.1%). Taking each college separately, high blood pressure was more prevalent among EMS, pharmacy, and nursing students (93.4, 90.5, and 84.5% respectively). A large majority of all groups of pharmacy, nursing, and EMS (90.5, 84, and 82%) students identified heart disease as one of the risk factors for stroke. While 85.2 % of EMS, 84.1% of pharmacy, and 72.8% of nursing students reported older age as the risk factor for stroke. Interestingly, only a small percentage of all group students reported that gender is also a risk factor for stroke. More details can be found in Table 2. In this study, 41.3 % of the pharmacy students reported a good level of knowledge than nursing (33.3%) and EMS (21.3%) students. The detailed descriptions of the individual knowledge score among the healthcare undergraduates were given in Figure 1.

**Table 2:** Participant's knowledge of risk factors of strokes (*n* = 205).

**Risk factors**	**Pharmacy (*n =* 63) *n* (%)**	**Nursing (*n =* 81) *n* (%)**	**EMS (*n =* 61) *n* (%)**	**Total (*n =* 205) *n* (%)**
**Diabetes**				
True	43 (68.3)	60 (74.1)	44 (72.1)	147 (71.7)
False	10 (15.9)	13 (16.0)	10 (16.4)	33 (16.1)
I Don't Know	10 (15.9)	08 (9.9)	7 (11.5)	25 (12.2)
**Hypertension**				
True	57 (90.5)	68 (84.0)	57 (93.4)	182 (88.8)
False	5 (7.9)	8 (9.9)	2 (3.3)	15 (7.3)
I don't know	01 (1.6)	5 (6.2)	2 (3.3)	8 (3.9)
**Hyperlipidemia**				
True	58 (92.1)	49 (60.5)	36 (59.0)	143 (69.8)
False	2 (3.2)	13 (16.0)	6 (9.8)	21 (10.2)
I don't know	3 (4.8)	19 (23.5)	19 (31.1)	41 (20.0)
**Heart disease**				
True	57 (90.5)	68 (84.0)	50 (82.0)	175 (85.4)
False	3 (4.8)	11 (13.6)	5 (8.2)	19 (9.3)
I don't know	3 (4.8)	2 (2.5)	6 (9.8)	11 (5.4)
**Alcohol**				
True	39 (61.9)	55 (67.9)	39 (63.9)	133 (64.9)
False	10 (15.9)	15 (18.5)	8 (13.1)	33 (16.1)
I don't know	14 (22.2)	11 (13.6)	14 (23.0)	39(19.0)
**Tobacco**				
True	44 (69.8)	48 (59.3)	42 (68.9)	134 (65.4)
False	9 (14.3)	15 (18.5)	7 (11.5)	31 (15.1)
I don't know	10 (15.9)	18 (22.2)	12 (19.7)	40 (19.5)
**Birth control pills**				
True	19 (30.2)	31 (38.3)	26 (42.6)	76 (37.1)
False	18 (28.6)	18 (22.2)	20 (32.8)	56 (27.3)
I don't know	26 (41.3)	32 (39.5)	15 (24.6)	73 (35.6)
**High red blood cell count**				
True	21 (33.3)	43 (53.1)	37 (60.7)	101 (49.3)
False	22 (34.9)	12 (14.8)	9 (14.8)	43 (21.0)
I don't know	20 (31.7)	26 (32.1)	15 (24.6)	61 (29.8)
**Older age**				
True	53(84.1)	59 (72.8)	52 (85.2)	164 (80.0)
False	6(9.5)	15 (18.5)	3 (4.9)	24 (11.7)
I don't know	4(6.3)	7 (8.6)	6 (9.8)	17 (8.3)
**Gender**				
True	31 (49.2)	35(43.2)	23 (37.7)	89 (43.4)
False	14 (22.2)	28(34.6)	24 (39.3)	66 (32.2)
I don't know	18 (28.6)	18 (22.2)	14 (23.0)	50 (24.4)
**Heredity or genetics**				
True	37 (58.7)	42 (51.9)	39 (63.9)	118 (57.6)
False	7 (11.1)	19 (23.5)	11 (18.0)	37 (18.0)
I don't know	19 (30.2)	20 (24.7)	11 (18.0)	50 (24.4)
**History of prior stroke**				
True	55 (87.3)	54 (66.7)	49 (80.3)	158 (77.1)
False	3 (4.8)	15 (18.5)	6 (9.8)	24 (11.7)
I don't know	5 (7.9)	12 (14.8)	6 (9.8)	23 (11.2)
**Lack of exercise**				
True	48 (76.2)	60 (74.1)	48 (78.7)	156 (76.1)
False	5 (7.9)	10 (12.3)	7 (11.5)	22 (10.7)
I don't know	10 (15.9)	11 (13.6)	6 (9.8)	27 (13.2)

Regarding the warning sign of stroke, the majority of the respondents 164 (80%) identified difficulty in speaking and understanding or slurred speech, while 163 (79.5%) identified dizziness and loss of balance. On the other hand, 159 (77.6%) students, followed by chest pain or heart palpitations and difficulty in walking respectively, reported blurred vision. Focusing only on one of the highest warning signs of stroke, when students were compared on that basis, pharmacy students represented the highest proportion of 85.7%, while others were EMS and Nursing (80.3 and 75.3%) (Table 3). Additionally, when the same approach was used concerning the loss of balance, pharmacy, dental, and medical students' results were comparable, whereas nursing students had the lowest proportion of 72.8%. While 86.9% of EMS students had a higher proportion of dizziness and a similar percentage was found in both groups pharmacy and nursing (~76.5%). More details can be found in Table 4.

**Table 3:** Participants responses toward warning signs of strokes.

**Warning signs**	**Pharmacy (*n =* 63) *n* (%)**	**Nursing (*n =* 81) *n* (%)**	**EMS (*n =* 61) *n* (%)**	**Total (*n =* 205) *n* (%)**
**Blurred vision in 1 or both eyes**				
True	48 (76.2)	60 (74.1)	51 (83.6)	159 (77.6)
False	3 (4.8)	8 (9.9)	3 (4.9)	14 (6.8)
I don't know	12(19.0)	13 (16.0)	7 (11.5)	32 (15.6)
**Chest pain or heart palpitations**				
True	50 (79.4)	63 (77.8)	46 (75.4)	159 (77.6)
False	8 (12.7)	9 (11.1)	8 (13.1)	25 (12.2)
I don't know	5 (7.9)	9 (11.1)	7 (11.5)	21 (10.2)
**Difficulty in speaking and understanding or slurred speech**				
True	54 (85.7)	61 (75.3)	49 (80.3)	164 (80.0)
False	3 (4.8)	14 (17.3)	8 (13.1)	25 (12.2)
I don't know	6 (9.5)	6 (7.4)	4 (6.6)	16 (7.8)
**Difficulty in walking**				
True	55 (87.3)	55 (67.9)	49 (80.3)	159 (77.6)
False	3 (4.8)	17 (21.0)	5 (8.2)	25 (12.2)
I don't know	5 (7.9)	9 (11.1)	7 (11.5)	21 (10.2)
**Dizziness**				
True	48 (76.2)	62 (76.5)	53 (86.9)	163 (79.5)
False	4 (6.3)	8 (9.9)	2 (3.3)	14 (6.8)
I don't know	11 (17.5)	11 (13.6)	6 (9.8)	28 (13.7)
**Loss of balance**				
True	54 (85.7)	59 (72.8)	50 (82.0)	163 (79.5)
False	4 (6.3)	10 (12.3)	5 (8.2)	19 (9.3)
I don't know	5 (7.9)	12 (14.8)	6 (9.8)	23 (11.2)
**Numbness or weakness of the face and or limb of the body**				
True	53 (84.1)	55 (67.9)	48 (78.7)	156 (76.1)
False	3 (4.8)	9 (11.1)	5 (8.2)	17 (8.3)
I don't know	7 (11.1)	17 (21.0)	8 (13.1)	32 (15.6)
**Severe headache with unknown cause**				
True	47 (74.6)	58 (71.6)	49 (80.3)	154 (75.1)
False	6 (9.5)	11 (13.6)	6 (9.8)	23 (11.2)
I don't know	10 (15.9)	12 (14.8)	6 (9.8)	28 (13.7)
**Shortness of breath**				
True	38 (60.3)	54 (66.7)	36 (59.0)	128 (62.4)
False	8 (12.7)	6 (7.4)	9 (14.8)	23 (11.2)
I don't know	17 (27.0)	21 (25.9)	16 (26.2)	54 (26.3)

**Table 4:** Participants responses toward management of stroke.

**Basic information**	**Pharmacy (*n =* 63) *n* (%)**	**Nursing (*n =* 81) *n* (%)**	**EMS (*n =* 61) *n* (%)**	**Total (*n =* 205) *n* (%)**
**I will call the ambulance**				
Yes	41 (65.1)	47 (58.0)	56 (91.8)	144 (70.2)
No	22 (34.9)	34 (42.0)	5 (8.2)	61 (29.8)
**I will give home remedies**				
Yes	7 (11.1)	9 (11.1)	2 (3.3)	18 (8.8)
No	56 (88.9)	72 (88.9)	59 (96.7)	187 (91.2)
**I will give the patient the first painkiller to control the pain**				
Yes	3 (4.8)	8 (9.9)	5 (8.2)	16 (7.8)
No	60 (95.2)	73 (90.1)	56 (91.8)	189 (92.2)
**I won't do anything to let the patient recover by him/herself**				
Yes	0 (0)	1 (1.2)	1 (1.2)	2 (1.0)
No	63 (100)	80 (98.8)	80 (98.8)	203 (99.0)
**I will take the patient to the hospital immediately**				
Yes	1 (1.2)	34 (42.0)	24 (39.3)	83 (40.5)
No	80 (98.8)	47 (58.0)	37 (60.7)	122 (59.5)

In the case of a suspected case of stroke, the majority of the students (70.2%) agreed that they would call an ambulance. On the other hand, 40.5% of them agreed to take the patient to the hospital immediately when the patient is suffering from a stroke. Detailed information about the management of stroke among undergraduates was given in Table 4.

**Figure 2:**
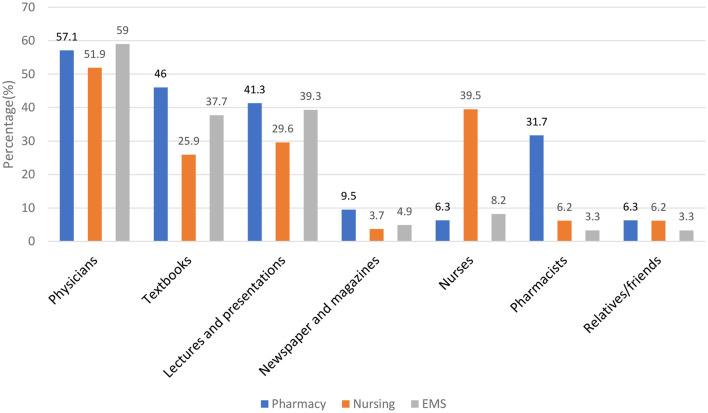
Source of information.

With regards to the source of information about stroke hundred and fourteen 114 (55.6%) reported physicians followed by lectures and presentations 74 (36.1%) and textbooks 73 (35.6%) respectively. More detailed information about the source of information for the stroke was given in Figure 2.

Table 5 shows the Association between the knowledge score of the participants concerning demographic characteristics of participants. We did not find any significant association between knowledge score and educational degree (*p* = 0.057). Similarly, the knowledge score of the undergraduates was not significantly associated with parents working in healthcare settings (*p* = 0.992). However, there was a significant association between knowledge score and year of study (*p* = 0.020) as shown in Table 5.

**Table 5:** Association between knowledge score of the participants concerning demographics.

**Participants characters**	**Number of respondents**	**Knowledge score**		***P*-value**
		**Good (*****N*** = **66; 32.2%)**	**Poor (*****N*** = **139; 67.8%)**	
**Educational degree**				0.049
Pharmacy	Count	26	37	
	% Within Educational degree	41.3%	58.7%	
	% Within knowledge levels	39.4%	26.6%	
Nursing	Count	27	54	
	% Within Educational degree	33.3%	66.7%	
	% Within knowledge levels	40.9%	38.8%	
EMS	Count	13	48	
	% Within Educational degree	21.3%	78.7%	
	% Within knowledge levels	19.7%	34.5%	
**Do any of your parents work in a healthcare setting**				0.992
Yes	Count	18	38	
	% Within parent's work in healthcare settings	32.1%	67.9%	
	% Within knowledge levels	27.3%	27.3%	
No	Count	48	101	
	% Within parent's work in healthcare settings	32.2%	67.8%	
	% Within knowledge levels	72.7%	72.7%	
**Level of education**				0.020
Third year	Count	27	81	
	% within the Level of education	25.0%	75.0%	
	% within knowledge levels	40.9%	58.3%	
Fourth-year	Count	39	58	
	% within the Level of education	40.2%	59.8%	
	% within knowledge levels	59.1%	41.7%	

## Discussion

This survey assessed the knowledge of Risk Factors and Warning Signs of Stroke among future healthcare professionals (pharmacy, nursing, and EMS) at KSU, Riyadh Saudi Arabia. The current findings reported 32.2% of the UHCS from KSU found knowledge about the stroke, while the majority of them reported poor knowledge. On the other hand, data on the health care undergraduates about the clinical presentation of stroke is limited, however, some studies exist on this issue, but most of the studies were conducted in other populations (11, 12, 24, 25). This study would add a significant contribution to enhancing the health care professionals, patients, and individuals' knowledge about stroke, thereby helping in some aspects of the management of the diseases in Saudi Arabia, and other countries and would serve as a reference for the much-needed upcoming studies. The research findings may also be used by healthcare and educational organizations to create effective training programs to increase the clinical presentation of stroke understanding by healthcare workers.

The current findings were inconsistent with previous findings (11–13, 24). For example, the previous study by Kankaya and Yesilbalkan among Nigerian nursing undergraduates reported 53.2% of the studied population were knowledgeable about risk factors, while 53.2% of them were knowledgeable about warning signs (53.8%) of stroke (11). However, another similar study reported 62.6% of the students were knowledgeable about various aspects of the stroke (24). It is commonly known that practicing healthcare professionals would be found to have good knowledge, followed by student professionals or the public. Even though knowledge may vary from study to study and may be influenced by many factors including the study method, the nature of respondents, and demographics.

A previous study by Alam et al. among university students of Dhaka evaluated the awareness about stroke and reported that 74.2% of the students identified stroke as a brain disorder (12). While in our findings 91.7% of the UHCS identified stroke as a brain disorder. Similarly, in another recent study in the United States, 50.1% of the students recognized stroke as a brain disorder (26). With regards to the meaning or definition of stroke, 91.7 % of UHCS in the current study recognized correctly as stroke occurs when blood flow to the brain is stopped. On the other hand, a similar previous study reported 36.7% of the medical students correctly identified that both thrombotic and hemorrhagic represent a stroke (13). Healthcare students' awareness of such important disease knowledge during their graduation would help at their practice site, which could save the lives of individuals who suffered or admired with history of stroke. Additionally, this research revealed that there were discrepancies in students' understanding of certain aspects of stroke, demonstrating the need for additional educational initiatives to raise students' awareness of various chronic diseases and their pathophysiology.

The current findings identified hypertension (88.8%) as a risk factor for stroke, followed by heart disease (84.5%), older age (80%), and history of prior stroke (77.1%). These results were similar to many previous studies conducted around the world (13, 24, 27). For instance, a previous study by al-Malki et al. identified high blood pressure followed by high cholesterol and smoking as the risk actors of stroke (13). In contrast, a study in southwestern Nigeria among undergraduate students concluded that hypertension (82.6%), old age (74.9%), hypercholesterolemia (42.8%), diabetes (35.9%) and smoking (27%) were the commonly identified stroke risk factors (24). Conversely, a recent study by Mirghani et al. (27) in Saudi Arabia reported that 90.4% of female medical students and 88.8 % of male medical students identified hypertension as a risk factor for stroke (27). Previous studies conducted among the public identified hypertension as the most common stroke risk factor in line with our findings (25). Furthermore, the American heart association and WebMD reported that constant stress is also another potential risk factor for stroke. The stress causes hypertension, which may cause constant strain on the heart arteries. When blood vessels are overinflated, too much force damages the walls of the arteries and makes them weaker. High blood pressure makes both main types of stroke more likely. Diabetes, high cholesterol, obesity, and older age were the vital factors that can cause a stroke (28, 29). Increased awareness about early signs of stroke can improve overall disease diagnosis and treatment, morbidity, and death rates.

In our study, according to students' perception toward warning signs of stroke, commonly identified symptoms were difficulty in speaking/understanding or slurred speech, dizziness and loss of balance, blurred vision, chest pain or heart palpitations, and difficulty in walking respectively. These findings were consistent with similar studies conducted in Saudi Arabia and other countries (11, 27, 30). Other reports revealed numbness or weakness, difficulty in understanding speech, trouble in speaking or seeing, and walking and headache are important signs and symptoms of stroke (31). According to our findings, more than half of the students believed that physicians were the most knowledgeable healthcare professionals to provide the source of information about stroke. While a previous study showed that textbooks (37.0%) and the internet (18.5%) were the most commonly cited resources (11). It is known that the best source of information about stroke and better interventions can only be provided by the physician.

Lastly, the action to be taken in case of stroke is vital during emergency cases. However, in a study by Almalki et al. (13) study, more than two-thirds (69.7%) of the students would call an ambulance and this was followed by driving to the nearest hospital (51.8%) and telling the patient's family member (47.8%). In addition, calling an ambulance (95%) was also the prime action among the nursing students which was reported by Kanaya et al. (11). Furthermore, a previous study conducted among university students reported (85.7 %) would take a patient to a hospital for any potential stroke, (12) while our study reported 70.2% reported calling an ambulance would be the first appropriate action. In this current research, we emphasize the importance of further studies that can evaluate the perceptions of HealthCare students. This study provides a good platform for others to conduct research within the domains.

In this study, the knowledge level of the stroke is significantly associated with the course, being pursued and the year of study. Studies examining the variation between knowledge of stroke and characteristics of UHCS are currently lacking. There have been some studies about the evaluation of knowledge of clinical presentations of stroke among prospective students (11–15), but those earlier studies did not look at the relationship between the knowledge score and the characteristics of undergraduates. The fact is that senior undergraduates consistently demonstrate a higher level of theoretical knowledge than juniors. Additionally, prior exposure to clinical knowledge during the graduation process (through a course, congress, seminar, etc.) may have affected this circumstance.

However, the current study has some limitations. Firstly, the results were based on a self-completed questionnaire, Secondly, the results were derived from a single institute in Saudi Arabia, therefore, the findings of this study cannot be generalized to the whole Saudi Arabia, studnets population. Thirdly, the study did not involve junior students as it was conducted among senior healthcare students of the university, given the more accessible access to students found while spreading the questionnaire. Despite these limitations, our study lays more emphasis on increasing the awareness toward knowledge of risk factors and warning signs/symptoms of stroke and its complications to make them more competent in raising public health.

## Conclusion

This study depicts that one-third of the undergraduate healthcare students were found to have good knowledge. The knowledge score was significantly higher among pharmacy undergraduates compared to nursing and EMS healthcare students. Furthermore, the knowledge was significantly associated with the year of study whereas there were no significant differences between educational degrees. Thus, health education programs might help the students to understand clinical presentations of stroke. Incorporating more advanced topics about stroke and various chronic diseases in clinical practice will undoubtedly enhance treatment outcomes, reduce adverse medication effects, and have a favorable impact on patient care in the future.

## Data availability statement

The original contributions presented in the study are included in the article/supplementary material, further inquiries can be directed to the corresponding author.

## Ethics statement

Ethical review and approval was not required for the study on human participants in accordance with the local legislation and institutional requirements. The patients/participants provided their written informed consent to participate in this study.

## Author contributions

WS, OQ, AB, EA, and MBAA: conceptualization of research and editing and review of manuscript. WS: data collection. OQ, AB, EA, and MBAA: data analysis and drafting the manuscript. All authors contributed to the article and approved the submitted version.
